# Genomic Alterations in Quadruple-Negative Breast Cancer Tumors

**DOI:** 10.3390/ijms27156654

**Published:** 2026-07-25

**Authors:** Carolina Jaliffa, Uwe Rogel, Cornelia Leo, Gad Singer

**Affiliations:** 1 Institute of Pathology, Kantonsspital Baden AG, Im Ergel 1, 5404 Baden, Switzerland; 2 Breast Center, Kantonsspital Baden AG, Im Ergel 1, 5404 Baden, Switzerland; cornelia.leo@ksb.ch

**Keywords:** breast cancer, TNBC, QNBC, AR, genomics, HRD, cancer-related pathways, molecular pathology

## Abstract

Triple-negative breast cancer (TNBC) lacking androgen receptor (AR) expression defines quadruple-negative breast cancer (QNBC), which is characterized by younger age at diagnosis, high Ki-67 index, and high genomic instability, however a comprehensive description of the genomic characteristics remains poorly defined. A total of 54 TNBC cases were categorized as TNBC with 100% AR expression (TNBC AR-100%) or QNBC, TNBC with 0% AR expression (TNBC AR-0%). Clinical, molecular, and genomic parameters, specifically pathogenic/likely pathogenic (P/LP) variants in homologous recombination repair (HRR) and cancer-related pathways were measured and analyzed. The QNBC cohort exhibited a high homologous recombination deficiency (HRD) score and a greater overall incidence of copy number variants (CNVs). QNBC harbored a higher mutation rate in TP53 and MYC signaling pathway than TNBC AR-100% tumors. P/LP variants corresponding to the HRR, PI3K/AKT, and RTK/RAS pathways were exclusively identified in QNBC. These results suggest that, at both molecular and genomic levels, the two groups are distinct, holding QNBC tumors more aggressive characteristics, genomic instability, and particular impairments in HRR and cancer-related pathways. In terms of actionability, these differences could potentially be leveraged through different combinations of therapies.

## 1. Introduction

Triple-negative breast cancer (TNBC) is a heterogeneous subgroup of breast carcinoma, characterized by the absence of estrogen receptor (ER) and progesterone receptor (PR) protein expression, as well as a lack of HER2 (ERBB2) gene amplification or protein overexpression. The presence of androgen receptor (AR) expression in TNBC enables further classification: TNBC with positive AR expression, or TNBC lacking AR expression, also known as quadruple-negative breast cancer (QNBC) [1]. QNBC is most frequently associated with a younger age at diagnosis and a higher Ki-67 index [2,3]; however, evidence regarding differences in prognosis and chemotherapy outcomes compared to TNBC patients with AR-positive expression remains controversial [4,5].

Despite significant advances in TNBC treatment over the past decade, survival rates for patients with advanced TNBC remain limited and are considerably lower than those for other types of breast cancer [6]. For TNBC patients with AR-positive tumors, a rational therapeutic strategy is the use of AR antagonists; in this sense, clinical trials investigating the use of AR inhibitors, either as monotherapy or in combination strategies, are currently underway [7,8]. However, QNBC patients—who represent the majority of TNBC cases—do not benefit from these potential therapeutic options. In the era of targeted therapies, genomic characterization of QNBC is essential for optimizing treatment strategies.

Although the genomic landscape of TNBC has been studied, comprehensive genomic data and specific pathogenic variants in homologous recombination repair (HRR) and cancer-related pathways for QNBC remain poorly defined. Previous research has shown that QNBC is characterized by genomic instability, including copy number alterations (CNAs) and deregulation of microRNAs (miRNAs) expression [9]. However, little is known about the specific mutation landscape of QNBC, which is crucial for testing novel anti-tumor agents and combination therapies. The aim of this study is to provide a comprehensive description of the genomic characteristics and potential actionable targets of TNBC patients in relation to AR expression, defining two groups: (1) TNBC with 100% AR (TNBC AR-100%) expression, and (2) QNBC, consisting of patients with TNBC lacking AR expression (TNBC AR-0%).

## 2. Results

### 2.1. Clinicopathological Characteristics of TNBC Patients Based on AR Expression

**Figure 1:**
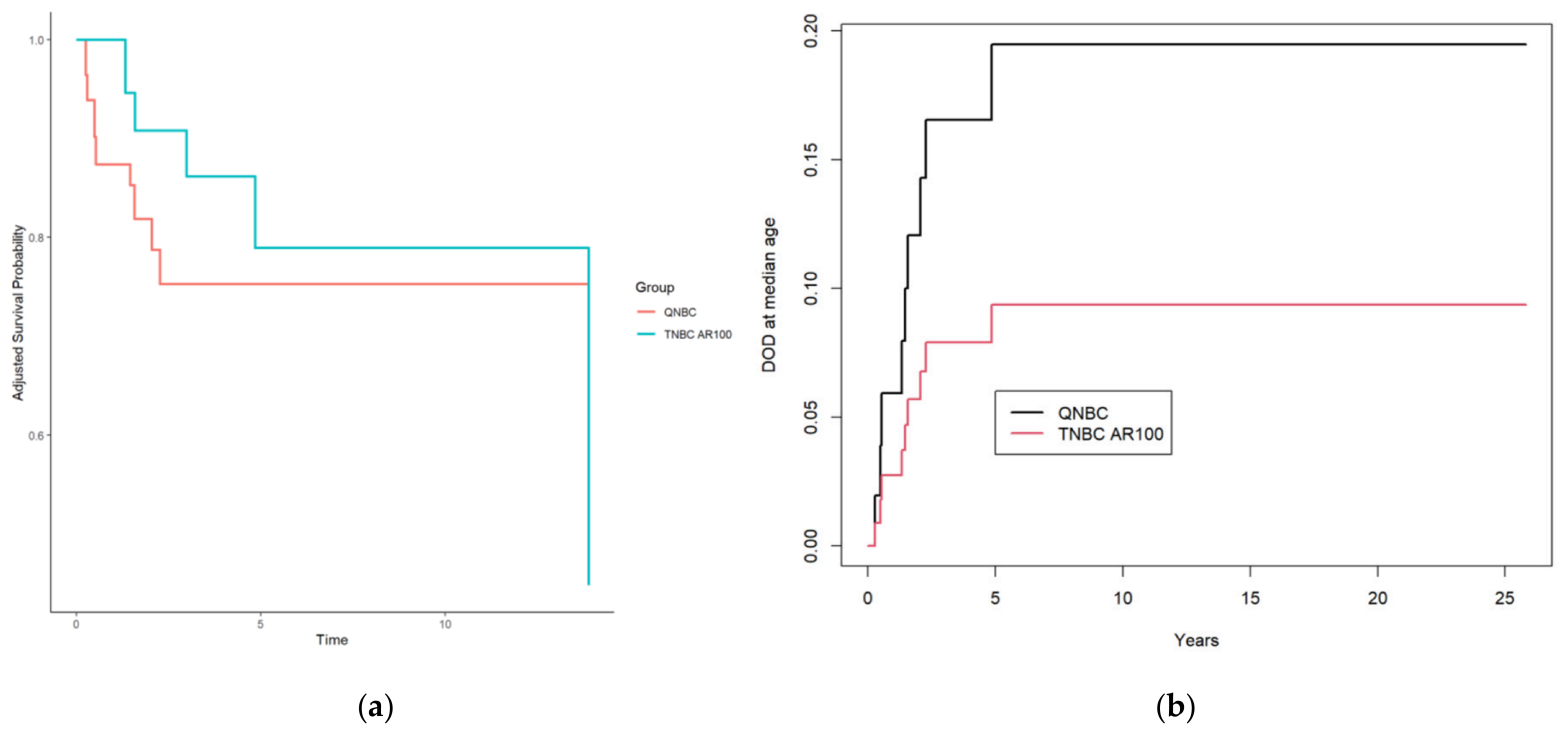
Survival outcomes in TNBC patients according to the percentage of AR expression. (**a**) Overall survival (OS) and (**b**) cumulative risk of cause-specific death in TNBC AR-100% vs. QNBC patients. DOD—death associated to TNBC diagnosis, test for proportional hazard gives no significant deviation (*p* > 0.05).

A total of 54 TNBC cases were evaluated and classified according to their AR expression in TNBC AR-100% (n = 16) and TNBC AR-0%, referred to as QNBC (n = 38). Table 1 summarizes the clinicopathological features of the TNBC cases included in this study. At diagnosis, QNBC patients were significantly younger than TNBC AR-100% patients (53 vs. 65 years, *p* = 0.007; Table S1). No differences were observed between the groups regarding the incidence of multifocal tumors or tumor laterality. Chemotherapy regimens in both neoadjuvant and/or adjuvant settings were administered at similar rates in both groups (86% for TNBC AR-100% and 94% for QNBC). Recurrences and metastasis were observed without significant differences between the groups during the follow-up period (Table 1 and Table S1). Differences in overall survival (OS) analysis, as well as cumulative risk of cause-specific death adjusted to age, were found to be non-significant across both groups (Figure 1).

Table 2 shows the pathological characteristics at histopathological and molecular levels of TNBC tumors according to the percentage of AR expression. Her2-low expression was detected in 63% of the TNBC AR-100% group and 42% of the QNBC group while Her2-0 expression was identified in 38% of the TNBC AR-100% set and in 58% of the QNBC group. Most patients displayed no special histological type (NST). Metaplastic histological subtype showed a higher incidence in the QNBC group and apocrine histological subtype in the TNBC AR-100% set (Table 2). QNBC tumors had a higher incidence of histopathological grade 3 at diagnosis than TNBC AR-100% (97% vs. 44%, respectively, *p* < 0.001). QNBC had an increased incidence of patients with high Ki-67 index (≥30%) compared to TNBC AR-100% (95% vs. 63% respectively, *p* = 0.0058). The TNBC cohort showed a higher incidence of low tumor-infiltrating lymphocytes (TILs) compared to the incidence of intermediate or high TILs, and this parameter was equal in both groups (Table 2 and Table S1). Collectively, these results indicate that QNBC tumors could be more aggressive at the molecular and histological levels than TNBC AR-100%.

### 2.2. Genomic Characterization of TNBC Tumors Related to AR Expression Levels

**Figure 2:**
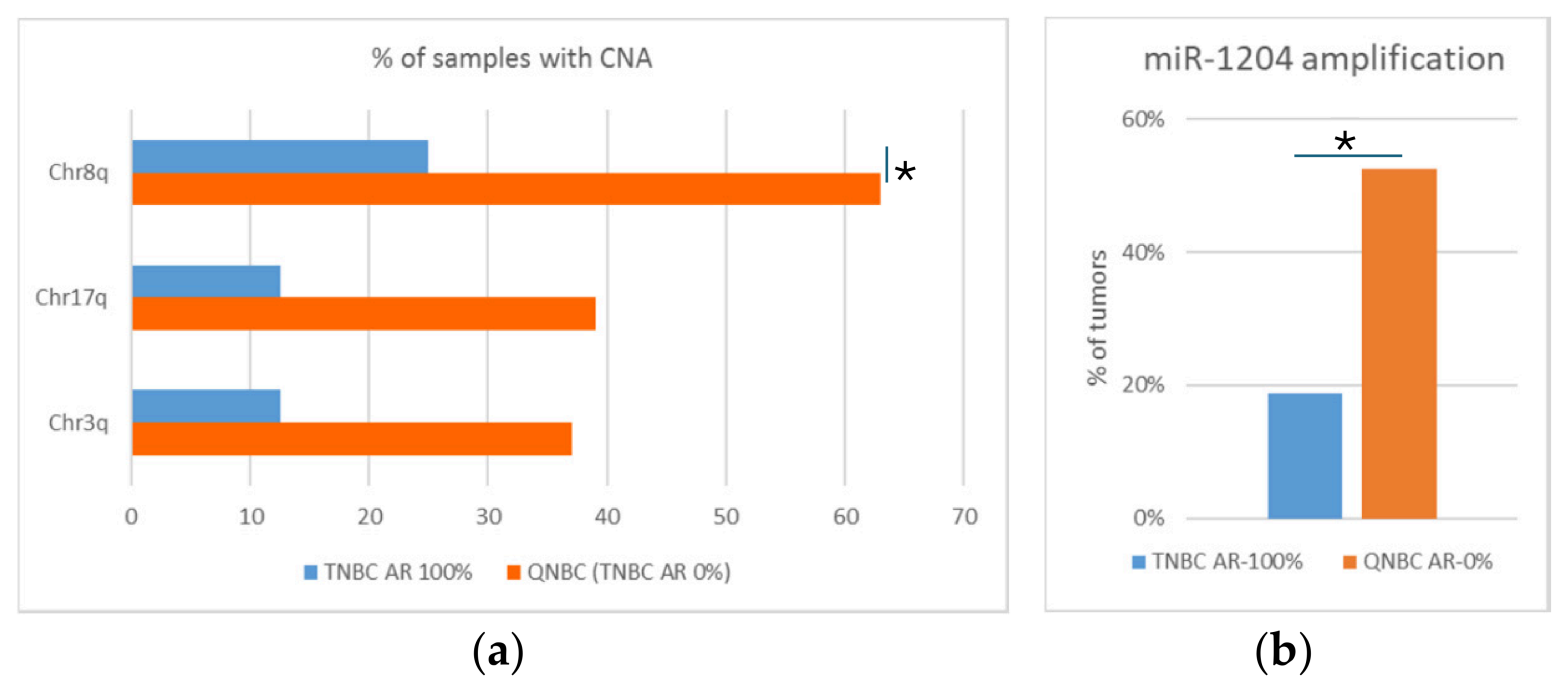
Frequency distribution alteration of CNVs and microRNA 1204 (miR-1204) in TNBC tumors associated with AR status. Frequency distribution of (**a**) Focal CNVs grouped by chromosome arm and (**b**) miR-1204 amplification in TNBC AR-100% vs. QNBC tumors; * *p* < 0.05.

The global genomic characteristics of the TNBC samples associated with AR expression included in this study are summarized in Table 3. TNBC samples showed microsatellite stable (MSS) to low microsatellite instability (MSI-L) in microsatellite status and low tumor mutation burden (TMB) values, without differences detected between the groups. Only one sample in TNBC AR-100% displayed high MSI (MSI-H), and one sample in QNBC displayed high TMB (TMB > 10 mutations per megabase (Mut/Mb) (Table 3 and Table S1). Homologous recombination deficiency (HRD) score was significantly higher in the QNBC set of samples compared to the TNBC AR-100% group (Table 3 and Table S1). The incidence of total copy number variants (CNVs) per tumor was significantly higher in QNBC than in TNBC AR-100% samples. In addition, the percentage of neoplasms harboring at least one CNV was significantly increased in QNBC in relation to TNBC AR-100% group (97% vs. 56%, respectively) (Table 3 and Table S1). Figure 2a shows the detailed total CNV frequency analyzed by chromosome arm. Most frequent focal CNVs were identified in 8q cytobands in both groups; the incidence of focal CNVs was significantly higher in QNBC tumors than in TNBC AR-100% samples (63% vs. 25%, respectively; *p* = 0.016). The incidence of focal CNVs in 17q and 3q cytobands was the next most frequent altered cytobands in the QNBC group (Figure 2a and Table S2). In addition, miRNA 1204 amplification was examined, as it has been reported to be differentially amplified in QNBC [9]. miRNA 1204 was amplified in 19% of the TNBC AR-100% samples compared to 53% of the QNBC group (*p* = 0.034, Figure 2b). These findings highlight a potential increased genomic instability in QNBC tumors than in TNBC AR-100%.

### 2.3. Differences in Pathogenic/Likely Pathogenic (P/LP) Variant Rate According to AR Status in TNBCs Through Homologous Recombination Repair (HRR) and Main Cancer Signaling Pathways

**Figure 3:**
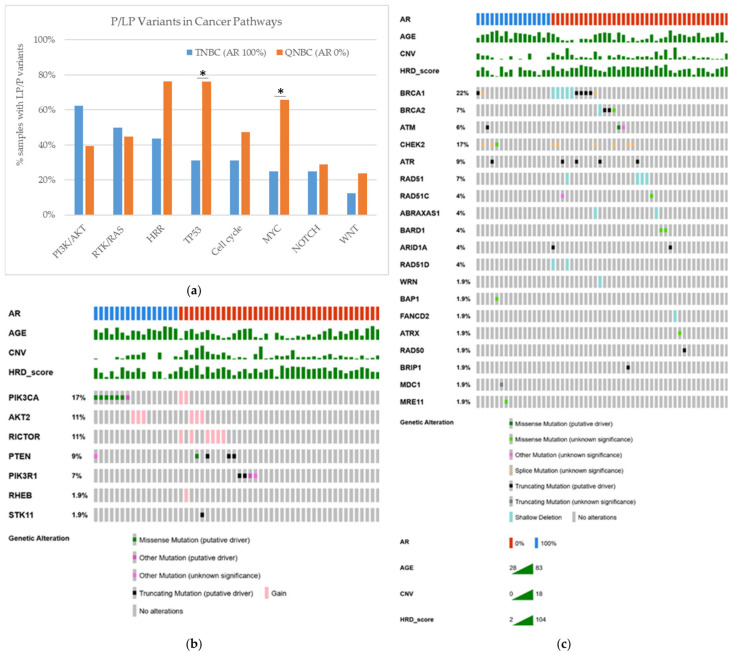
Alterations in pathogenic/likely pathogenic (P/LP) variants proportions according to AR status in TNBCs through homologous recombination repair (HRR) and main cancer signaling pathways * *p* < 0.05. (**a**) Proportion of samples showing at least one P/LP variant in the corresponding pathway in the TNBC cohort according to AR status in the PI3K/AKT pathway, RTK/RAS pathway, HRR pathway, TP53, Cell cycle pathway, MYC pathway, NOTCH pathway and WNT signaling pathway. Detailed distribution of P/LP variants in (**b**) HRR pathway and (**c**) PI3K/AKT pathway according to AR status in TNBCs.

Differences in P/LP variants between the groups were analyzed in the context of HRR and main signaling pathways involved in cancer (Figure 3, Table S3). Figure 3a shows a significant differential distribution of P/LP variants in the TP53 and MYC pathway between the studied groups. Affected samples with TP53 P/LP variants were significantly more prevalent in the QNBC group than in the TNBC AR-100% (76% vs. 31%, *p* = 0.032, respectively; Figure 3a). In the MYC pathway (MYC, MXD4, MGA, MLX), a significantly higher rate of P/LP variants was found in the QNBC group compared to the TNBC AR-100% set (66% vs. 25%, *p* = 0.032, respectively; Figure 3a). In the HRR pathway, the prevalence of tumors with P/LP variants was higher in the QNBC group than in the TNBC AR-100% set, but not statistically significant. Detailed distribution of these variants in the HRR pathway showed that BRCA1, ATM, CHEK2 and ATR mutations were found in both groups while BRCA2, RAD51, RAD51C, ABRAXAS1, BARD1, ARID1A, RAD51D, WRN, BRIP1, FANCD2, ATRX and RAD50 mutations were found only in QNBC tumors (Figure 3b). In the PI3K/AKT pathway, the percentage of samples presenting at least one P/LP variant was 63% in TNBC AR-100% compared to 39% in QNBC. Although the proportions of affected samples between both groups were statistically not significantly different, missense mutations in PIK3CA were exclusively present in the TNBC AR-100% group, while P/PL variants in RICTOR and PIK3R1 were only observed in the QNBC group. PTEN variants were mostly found in the QNBC group (Figure 3c). In the RTK/RAS pathway, the proportions of samples presenting P/LP variants were similar and over 40% in both groups. P/LP variants in cell cycle, NOTCH and WNT signaling pathways showed no differences across the groups. The detailed distribution of P/LP variants was identified for every pathway (Figure S1 and Table S3). These results suggest that AR status in TNBCs could be linked to distinct HRR and cancer-related pathways impairments as well as to specific LP/P genetic variants.

### 2.4. Comprehensive Genomic Summary in TNBC Patients According to AR Status

**Figure 4:**
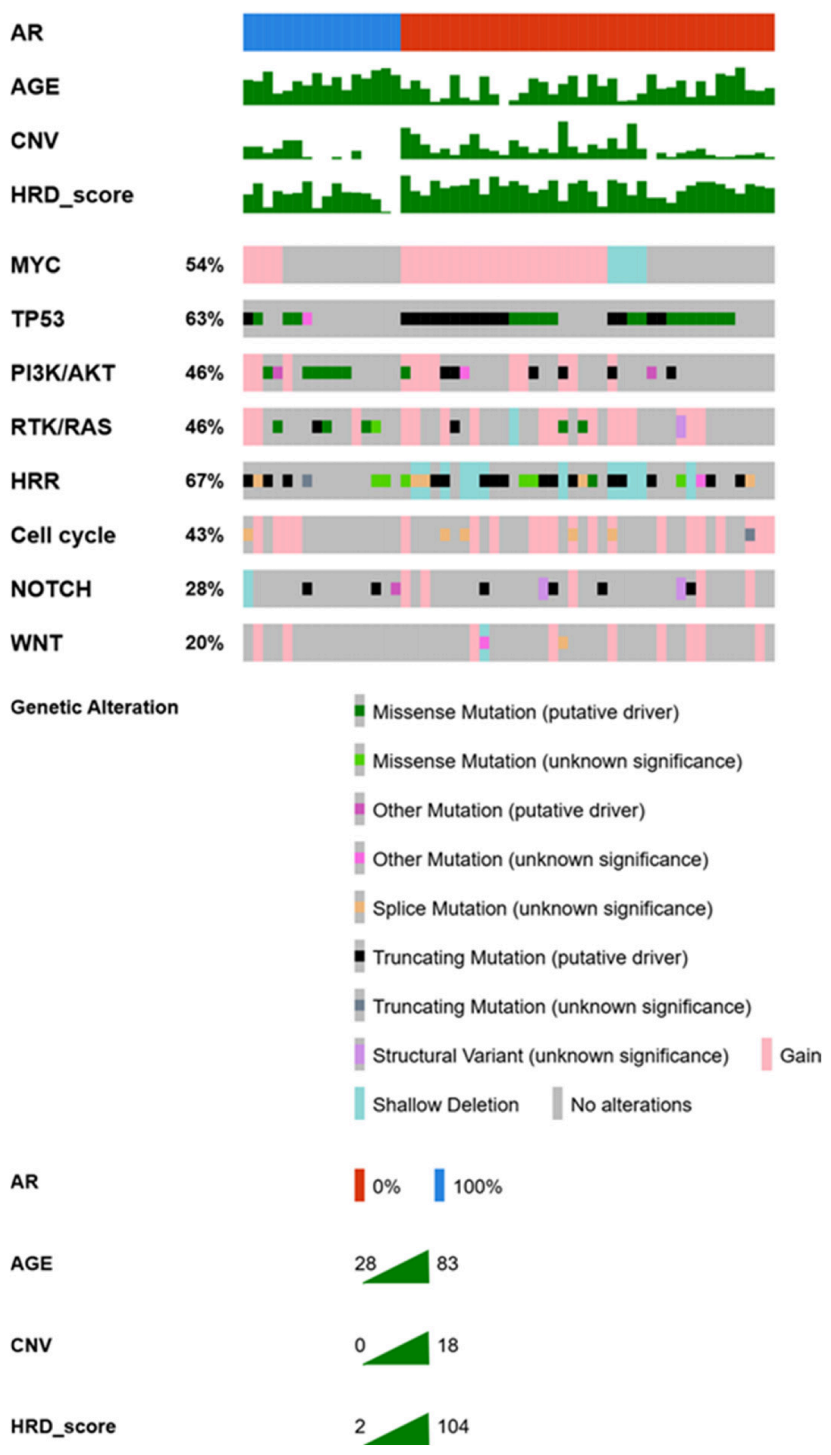
Comprehensive genomic summary of pathway alteration in the TNBC cohort stratified by AR expression, showing patients with AR-100% in blue and QNBC patients in red (TNBC AR-0%).

Figure 4 represents the overlap of genomic alterations in the TNBC AR-100% and QNBC cohorts. A combination of TP53 mutations and MYC pathway alteration was observed in 53% (20/38) of QNBC tumors, while only 13% (2/16) showed this overlap in TNBC AR-100% neoplasms (53% QNBC vs. 13% TNBC AR-100%; *p* = 0.006). Additionally, patients with wild-type TP53 and MYC pathways were more common in the TNBC AR-100% group than in QNBC (56% vs. 11%; *p* = 0.001, respectively). The incidence of HRR impairment, assessed by HRD score (HRD score ≥ 56) and/or the presence of a P/LP variant in the HRR pathway, was similar in both groups (81% for TNBC AR-100% vs. 92% for QNBC). Figure 4 also shows that most patients exhibit another molecular target in the cell cycle, the NOTCH and/or WNT cancer-related pathways, which may provide insight into a personalized medical context. The overlapping alterations underscore the complexity of treatment selection in TNBC, indicating that although overall HRR impairments are comparable across groups, treatment strategies could be tailored based on additional genomic differences. Collectively, these results demonstrate that in TNBC, identifying molecular targets is crucial for optimizing anticancer therapy combinations in relation to available therapeutic targets.

## 3. Discussion

Among these 54 TNBC cases classified based on either 100% or 0% AR expression, QNBC presented at diagnosis with a younger age, as well as higher histopathological grade and Ki-67 index, compared to TNBC AR-100% patients. The QNBC cohort exhibited a high HRD score and a greater overall incidence of total CNVs. Focal CNVs in the 8q cytobands include MYC and miR-1204 amplification. P/LP variants affecting cancer-specific pathways were comprehensively described, revealing that QNBC harbored a higher mutation rate in TP53 and MYC signaling pathways than TNBC AR-100% tumors. In addition, specific P/LP variants corresponding to the HRR, PI3K/AKT, and RTK/RAS pathways were exclusively found in QNBC. These results confirm clinico-pathological and histopathological differences and suggest that, at both molecular and genomic levels, the QNBC and TNBC groups are distinct. These differences between QNBC and TNBC groups could potentially be leveraged through different combinations of therapies.

### 3.1. Age at Diagnosis, Histopathology, and OS in QNBC Patients

Our study demonstrated that the QNBC cohort was diagnosed at a younger age compared to the TNBC AR-100% group, consistent with recent research involving QNBC patients with diverse genetic backgrounds [10,11,12,13]. Our results revealed a significantly higher Ki-67 index and higher histological grade in the QNBC cohort. Supporting our findings, increased Ki-67 index and histological grade were recently reported in both European [14] and non-European populations [10,13,15] for AR-negative TNBCs. In addition, we found no significant differences in OS or cumulative risk of cause-specific death, aligning with three other meta-analyses—including overlapping datasets—which reported no significant association between AR status and OS in TNBC patients [16,17,18]. Collectively, these results suggest that QNBCs exhibit more aggressive characteristics at the histopathological level, regardless of AR cutoff or genetic background. Nevertheless, this increased aggressiveness for QNBC is not reflected in OS, as might be expected. This paradox may be explained by the opposing influences of patient age and the differential and/or low effect of the anticancer treatments used (or not) on the OS.

### 3.2. Key Global Genomic Parameters in QNBC Tumors

This study demonstrates that QNBC tumors had a mean value of 4.8 Mut/Mb, which was not significantly different from the TNBC AR-100% group, and is consistent with median TMB values reported in European (1.52 Mut/Mb) [19] and Chinese (4.1 Mut/Mb) [20], (6.96 Mut/Mb) [21] TNBC cohorts. The MSS observed in both QNBC and TNBC cohorts confirms previous findings in TNBC [20,22] and now extends them to QNBC. Regarding AR expression, to our knowledge, this is the first time TMB and MSI values for QNBC have been clearly presented. Our results show that the incidence of total CNV per patient was higher in QNBC samples, consistent with earlier reports [9]. Furthermore, we found that focal CNVs were more frequent in 8q cytobands in QNBC. The partial agreement with Bhattarai et al. may reflect differences in methodology and population genetic background. Focal CNVs in the 8q cytobands include not only MYC amplification but also miR-1204 amplification, which is involved in cancer progression and may influence chemotherapeutic sensitivity [23]. Our study indicates that QNBC tumors had a higher HRD score compared to TNBC AR-100%. Supporting our findings, a low HRD score was reported in two TNBC-positive-AR/luminal AR cohorts compared to TNBC negative-AR/non-luminal AR sets, which may be comparable to our QNBC group [24,25]. Altogether, the increased HRD score, and CNV incidence observed in the QNBC tumor cohort suggest that the QNBC subtype is more genomically unstable than TNBC AR-100% tumors.

### 3.3. Genetic Variants in HRR and Cancer-Related Pathways

TNBCs have been examined from various perspectives regarding their subtypes and mutational landscape, both of which continue to evolve. Here, we present an approach focusing on AR expression and alterations in HRR and cancer-related pathways. In this study, we observed a significant increase in TP53 and MYC pathway alterations in QNBCs compared to TNBC AR-100%. Our findings are consistent with previous reports of TP53 and MYC pathway changes in TNBC cohorts, as well as differences between TNBC molecular subtypes [26,27,28]. Furthermore, lower rates of MYC gains and TP53 mutations have been noted in apocrine TNBCs, which exhibit elevated AR expression and are therefore comparable with our TNBC AR-100% group; while basal-like TNBCs, potentially comparable with our QNBC group, show increased TP53 mutation rates [29], in line with our results. The incidence of amplified variants in the PIK3CA and RTK/RAS pathways described here aligns with earlier findings that genes from these pathways (PIK3CA, KRAS, BRAF, FGFR1, KIT, and PDGFRA) are amplified but not commonly mutated in basal-like TNBC cancers [26]. Notably, our study shows that these amplified variants were found almost exclusively in QNBCs. PTEN variants found in the QNBC group were mostly truncating mutations, which is in line with the results found previously at expression levels, but at metastasis or recurrent sites [30]. DNA damage response-related pathways have been reported to be altered across TNBC molecular subtypes [21], differences in relation to AR status were not clear. In this study, we found exclusive mutations in BRCA2, RAD51, RAD51C, ABRAXAS1, BARD1, ARID1A, RAD51D, WRN, BRIP1, FANCD2, ATRX, and RAD50 in QNBC tumors. Although further research is needed to confirm these observations, QNBC tumors harbor increased and/or exclusive LP/P variants in HRR and cancer-related pathways. LP/P variants identification in TNBC AR-100% group and QNBC is essential because it could be related to differential tumor development and directly impact therapy decisions.

### 3.4. Potential Clinical Actionability in QNBCs

Interpreting the results in terms of therapeutic targets and/or preventing therapy resistance development is challenging due to overlapping of genomic features in the same patient, particularly HRR and cancer-related pathways alterations. In addition, concurrent TP53 and MYC pathway alterations in QNBC suggest high proliferation and genomic instability, consistent with an aggressive cancer phenotype and potential therapy resistance. A high HRD score is associated with the effectiveness of PARP inhibitors, in the context of concurrent TP53 and MYC pathway alterations, mostly occurring in the QNBC patients, it could influence the effectiveness of PARP inhibitors therapy [31]. Altogether, these findings suggest that more studies are necessary in this direction to achieve better clinical results in QNBC patients, probably weighing multiple anti-cancer approaches to treatment. In this sense, the ESMO Scale for Clinical Actionability of molecular Targets [32] could offer alternative therapeutic options for these patients, providing a framework to connect DNA alterations with specific targeted therapies. Beyond PARP-inhibitor eligibility conferred by HRD status, several of the P/LP variants identified in this cohort correspond to potential pathways with emerging or established targeted agents. In the PI3K/AKT pathway, PTEN, RICTOR, and PIK3R1 alterations are of particular interest given that the pan-AKT inhibitor capivasertib, used with paclitaxel in first-line metastatic TNBC, showed significantly longer PFS and OS [33]. For the concurrent TP53/MYC alterations, no direct inhibitor exists, but TP53-mutant tumors depend on the ATR-CHK1-WEE1 axis for checkpoint control, and the WEE1 inhibitor adavosertib combined with cisplatin has shown clinical activity in metastatic TNBC [34]; BET inhibitors targeting BRD4-MYC super-enhancer activity are also in early clinical development for TNBC [35]. Taken together, the pathway alterations enriched in QNBC potentially map onto an expanding, if still maturing, landscape of targeted and combination therapies.

Study limitations include the relatively small sample size, which was partly due to the exclusion of the patient population with intermediate AR expression; this could allow a clearer interpretation of the results obtained in the QNBC group. Additionally, genetic background information as well as the menopausal status of the patients were not available in the medical records, limiting the conclusions of our work. Finally, the retrospective and descriptive nature of this study may require confirmation in clinical settings using larger patient cohorts, but nonetheless provides an innovative setting and arguments to test new hypotheses.

Our aim was to study QNBC tumors compared to TNBC AR-100% to obtain a clearer distinction between these two groups, particularly regarding genomic characteristics. The QNBC group was characterized by younger patients, higher histological grade, elevated Ki-67 index, increased HRD score, and greater incidence of CNVs per patient. Furthermore, a higher incidence of TP53 mutations, increased MYC pathway alterations, and specific P/LP variants involving the PI3K/AKT, HRR, and RTK/RAS pathways were found only in QNBC. This study provides valuable data supporting the hypothesis that TNBC AR-100% and QNBC are different groups from a genomic perspective and therefore distinct anti-cancer therapy combination may be relevant for the successful treatment of the TNBC patients according to AR status.

## 4. Materials and Methods

### 4.1. Patients and Study Design

This retrospective and exploratory study included 54 cases of TNBC that underwent surgery at our institution. Tissue samples were collected during the initial surgical procedure or, in three cases, during local recurrence. All carcinomas were classified as TNBC based on histopathological evaluation in the routine diagnostic setting and subsequently reviewed by an experienced pathologist (G.S.) for this study, according to established international histopathological criteria. After confirming AR expression, TNBCs were further categorized as either TNBC AR-100% or as QNBC if AR expression was absent (TNBC AR-0%). The reason for selecting only AR-0% and AR-100% cases was to reduce clinical and biological heterogeneity. Furthermore, the cutoff for AR expression could act as a confounding factor, as there remains controversy over the threshold for classifying AR expression as positive.

Inclusion criteria for this study were: (1) medical diagnosis of ductal invasive breast cancer; (2) confirmation of TNBC by histopathological ER and PR score of 0% and absence of HER2 (ERBB2) amplification, determined by fluorescence in situ hybridization (FISH) and immunohistochemical assay; (3) AR expression of either 0% or 100%; and (4) availability of formalin-fixed, paraffin-embedded (FFPE) tissue. Cases with AR expression levels between 1–90% were excluded. Clinical data (including age at TNBC diagnosis, tumor laterality, multifocal or multicentric tumor, histopathological grade, follow-up time, anticancer therapy setting, recurrence and metastasis occurrence) and molecular data (such as Her2 protein status, HER2 amplification level by FISH, and Ki-67 index) were collected from hospital records (Table S1). Her2 protein expression, assessed by immunohistochemistry, was distinguished as Her2-low (1/2) or Her2-0. TILs were evaluated according to the International TILs Working Group guidelines [36]. The percentage of TILs on H&E-stained biopsy sections was reported as low (0–10%), intermediate (20–40%), or high (>50%).

This study was approved by the Ethics Committee of Northwestern Switzerland. Individual informed consent was waived due to the retrospective nature of the study (2021-01393).

### 4.2. Next Generation Sequencing (NGS) and Data Processing

Tumor areas were pre-marked by an experienced pathologist (G.S.) on an H&E- stained section and macro-dissected form FFPE slices. Total nucleic acid (TNA) from tumor areas was isolated using the Maxwell^®^ RSC RNA FFPE kit on the Maxwell^®^ RSC instrument (Promega, Madison, WI, USA) according to the manufacturer’s instructions. DNA was quantified using the QuantiFluor^®^ ONE dsDNA kit (Promega, Madison, WI, USA) with a Quantus™ fluorometer (Promega, Madison, WI, USA).

According to the manufacturer’s instructions, a total of 100 ng of each TNA sample were used to run DNA and RNA libraries with the QIAseq Multimodal HC Panel assay (QIAGEN Science, Germantown, MD, USA). Purified libraries were quantified as described above, normalized to 4 nM, pooled, and denatured to obtain a final concentration of 0.8 pM. The pooled libraries were sequenced by the Illumina NextSeq 550Dx platform (Illumina Inc., San Diego, CA, USA). The quality of sequencing runs was assessed using Illumina Sequencing Analysis Viewer (Illumina Inc., San Diego, CA, USA). A detection limit of 10% variant allelic frequency, a minimum of 50 reads and quality Q30 were used, and all synonymous variants were excluded. NGS data analysis was performed through the QIAseq Multimodal Data Analysis Portal to detect single-nucleotide variants, insertions–deletions, CNVs and fusions in 523 genes, including exon-level interpretation. CNVs for each target gene were indicated as either amplification or deletion depending on the fold-change value, over the defined thresholds. MSI status was determined based on the percentage of unstable sites out of the total number of successful microsatellite sites analyzed, defining three categories: MSS status for unstable loci < 15%; MSI-L for unstable loci between 15–40%; and MSI-H for unstable loci > 40%. The TMB for each sample was calculated by considering the number of non-synonymous eligible single nucleotide variants by the size of the successfully sequenced panel (1.44 Mb total panel size) and was expressed as the number of somatic Mut/Mb. NGS data assessment and interpretation were supported by QIAGEN Clinical Insight (QIAGEN Science, Germantown, MD, USA). Variants annotated as P/LP were included in this study. Variants of uncertain significance (VUS) were manually curated and retained if the alteration was annotated in OncoKB™ (https://www.oncokb.org/; last accessed on 17 July 2026) [37,38] as oncogenic/likely oncogenic or in ClinVar (https://www.ncbi.nlm.nih.gov/clinvar/; last accessed on 17 July 2026) [39] as P/LP variants. P/LP and VUS CNVs were used to calculate total CNVs.

To quantify the HRD score, QIAseq Targeted DNA Custom Panel (QIAGEN Science, Germantown, MD, USA) was used. Input was 40 ng of TNA from FFPE samples. HRD score was calculated as the weighted sum of the number of telomeric allelic imbalances, large-scale transitions, and long regions of loss of heterozygosity. The calculations were based on identified CNV regions and variant frequencies in a sample. The HRD score cut-off was set at ≥56; scores above this were considered deficient in homologous recombination.

Retained variants were assigned to an oncogenic signaling pathway [40] or to the HRR pathway [41,42,43]. For every signaling pathway analyzed, the following genes were included: PI3K/AKT signaling pathway: AKT1/2/3, INPP4B, MTOR, PIK3CA/B, PIK3R1/2/3, PPP2R1A, PTEN, RHEB, RICTOR, RPTOR STK11, and TSC1/2; NOTCH signaling pathway: ARRDC1, CNTN6, CREBBP, CUL1, DNER, EP300, FBXW7, HES-X, HEY-X, JAG2, KAT2B, KDM5A, MAML3, NCOR1/2, NOTCH1/2/3/4, NOV, PSEN2 and SPEN; RTK/RAS signaling pathway: ABL1, ALK, ARAF, BRAF, CBL, EGFR, ERBB2/3/4, ERRFI1, FGFR1/2/3/4, FLT3, HRAS, IGF1R, JAK2, KIT, KRAS, MAP2K1/2, MAP3K1, MET, NF1, NRAS, NTRK1/2, NTRK3, PDGFRA, PTPN11, RAC1, RAF1,RASA1, RIT1, RET, ROS1 and SOS1; WNT signaling pathway: AMER1, APC, AXIN1/2, CTNNB1, DKK1/2/3/4, GSK3B, LRP5/6, RNF43, SFRP1, TCF7, TCF7L1/2, TLE1/2/3/4, WIF1 and ZNRF3; TGF-β pathway: ACVR2A/1B, SMAD2/3/4, TGFBR1/2; Cell cycle pathway: CCND1/2/3, CCNE1, CDK2/4/6RB1, CDKN1A/B, CDKN2A/B/C, E2F1/3 and RB1; HIPPO signaling pathway: CRB1/2, CSNK1E/D, DCHS1/2, FAT1/2/3/4, LATS1/2, MOB1A/B, NF2, PTPN14, SAV1, STK3/4, TAZ, TAOK1/2/3/, TEAD2, WWC1 and YAP1; MYC signaling pathway: MAX, MGA, MLX, MLXIP, MLXPL MNT, MXD1/3/4, MXI1, MYC, MYCL, and MYCN; NRF2 signaling pathway: CUL3, KEAP1 and NFE2L2; TP53 gene; HRR pathway: ABRAXAS1, ARID1A, ATM, ATR, ATRX, BAP1, BARD1, BLM, BRCA1/2, BRIP1, CHEK1/2, FANCA/C/CI/D2/E/F/G/L/M, MDC1, MRE11A, NBN, NBS1, PALB2, RAD50, RAD51, RAD51B/C/D, RAD52 and WRN. To visualize and analyze P/LP variants for every cancer pathway, OncoPrinter (https://www.cbioportal.org/oncoprinter last accessed on 17 July 2026) was used [44,45,46].

### 4.3. Statistical Analysis

In this exploratory study, parameters were compared between groups using the Kruskal–Wallis test for continuous variables and Fisher’s exact test for categorical variables. Multistate competing risk analysis was performed to estimate the cumulative risk of dying from TNBC, using time in years from the date of initial diagnosis to the date of TNBC-related death or the last available follow-up. Results were adjusted for age kipping constant at the overall median value. Statistical analyses were performed using R version 4.4.3. The False Discovery Rate adjustment was applied to the *p*-values. All reported *p*-values are two-sided, and the significance level was set at *p* < 0.05.

## Figures and Tables

**Table 1:** Clinicopathological characteristics of triple-negative breast cancer (TNBC) patients according to the percentage of androgen receptor (AR) expression.

	TNBC	AR-100%	QNBC	(TNBC AR-0%)	*p*
Number of patients, n	16		38		
Age at TNBC diagnosis, years, mean, (range)	65	(46–82)	53	(28–83)	0.007
Tumor laterality, n (%)					ns
Left	8	(50)	16	(42)	
Right	8	(50)	20	(53)	
Bilateral	0	(0)	2	(5)	
Multifocal tumors, n (%)	4	(25)	4	(11)	ns
Administrated Chemotherapy ^a^, n (%)	12 out of 14	(86)	33 out of 35	(94)	ns
Recurrence ^b^, n (%)	2 out of 14	(14)	4 out of 31	(13)	ns
Metastasis ^b^, n (%)	4 out of 14	(29)	9 out of 31	(29)	ns
Follow up, years (range)	5	(0–15)	7	(0–26)	ns

TNBC with 100% AR expression—TNBC AR-100%; TNBC lacking AR expression—TNBC AR-0%; Quadruple-negative breast cancer—QNBC); ^a^ neo-adjuvant and/or adjuvant chemotherapy data from 5 cases out of the entire cohort were not available; ^b^ recurrence and metastasis data from 9 cases out of the entire cohort were not available; ns: not significant.

**Table 2:** Histopathological and molecular pathological characteristics of TNBC tumors according to the level of AR expression.

	TNBC	AR-100%	QNBC	(TNBC AR-0%)	*p*
Number of patients, n	16		38		
Her2 protein detection, n (%)					
Her2-low	10	(63)	16	(42)	ns
Her2-0	6	(38)	22	(58)	
Histopathological type, n (%)					
NST	9	(56)	28	(74)	ns
AP	6	(38)	0	(0)	
ME	1	(6)	5	(13)	
others	2	(13)	5	(13)	
Histopathological grade, n (%)					
G1–G2	9	(56)	1	(3)	<0.0001
G3	7	(44)	37	(97)	
Proliferation, n (%)					
Ki-67 index ≥30%	10	(63)	36	(95)	0.0058
Ki-67 index ˂30%	6	(38)	2	(5)	
TILs, n (%)					
low	11	(69)	24	(63)	ns
intermediate	2	(13)	8	(21)	
high	3	(19)	6	(16)	

NST—Not Special Type; ME—metaplastic; AP—apocrine; others histological types—neuroendocrine, micropapillary, medullar, lobular, small cell lung cancer; TILs—Tumor-Infiltrating Lymphocytes; ns—not significant.

**Table 3:** Global genomics alterations of TNBC tumors associated with AR expression.

	TNBC	AR-100%	QNBC	(TNBC AR-0%)	*p*
Number of patients, n	16		38		
MSS/MSI-L, n (%)	15	(94)	38	(100)	ns
TMB (Mut/Mb), mean (range)	2.3	(0.0–6.3)	4.8	(0.0–50.6)	ns
HRD score, mean (range)	49	(2–88)	71	(20–104)	0.005
Total CNVs per sample, mean (range)	2.8	(0–9)	5.4	(0–18)	0.038
Tumors with CNVs, n (%)	9	(56)	37	(97)	<0.001

Microsatellite stable (MSS); microsatellite instability (MSI) low MSI (MSI-L); tumor mutation burden (TMB); mutation per megabase (Mut/Mb), homologous recombination deficiency (HRD); copy number variants (CNVs); ns—not significant.

## Data Availability

The original contributions presented in this study are included in the article/Supplementary Materials. Further inquiries can be directed to the corresponding authors.

## Supplementary Materials

The following supporting information can be downloaded at: https://www.mdpi.com/article/10.3390/ijms27156654/s1.

## Abbreviations

The following abbreviations are used in this manuscript:

AR	androgen receptor
CNVs	copy number variants
ER	estrogen receptor
FFPE	formalin-fixed, paraffin-embedded
FISH	fluorescence in situ hybridization
HRD	homologous recombination deficiency
HRR	homologous recombination repair
miRNA	microRNA
MSI	microsatellite instability
MSI-H	high MSI status
MSI-L	low MSI status
MSS	microsatellite stable
Mut/Mb	mutation per megabase
NGS	next generation sequencing
P/LP	pathogenic/likely pathogenic
PR	progesterone receptor
QNBC	quadruple-negative breast cancer
TMB	tumor mutation burden
TNA	total nucleic acid
TNBC	triple-negative breast cancer
TNBC AR-100%	TNBC with 100% positive immunostaining for AR expression
TNBC AR-0%	TNBC with 0% positive immunostaining for AR expression
