# Supplementary material for: Genomic Alterations in Quadruple-Negative Breast Cancer Tumors
Source: Int J Mol Sci. 2026 Jul 25;27(15):6654. doi: 10.3390/ijms27156654 (PMC13466450; doi:10.3390/ijms27156654)
Supplement: Supplementary file 1 [file ijms-27-06654-s001.zip › Figure S1 revised2.pdf]

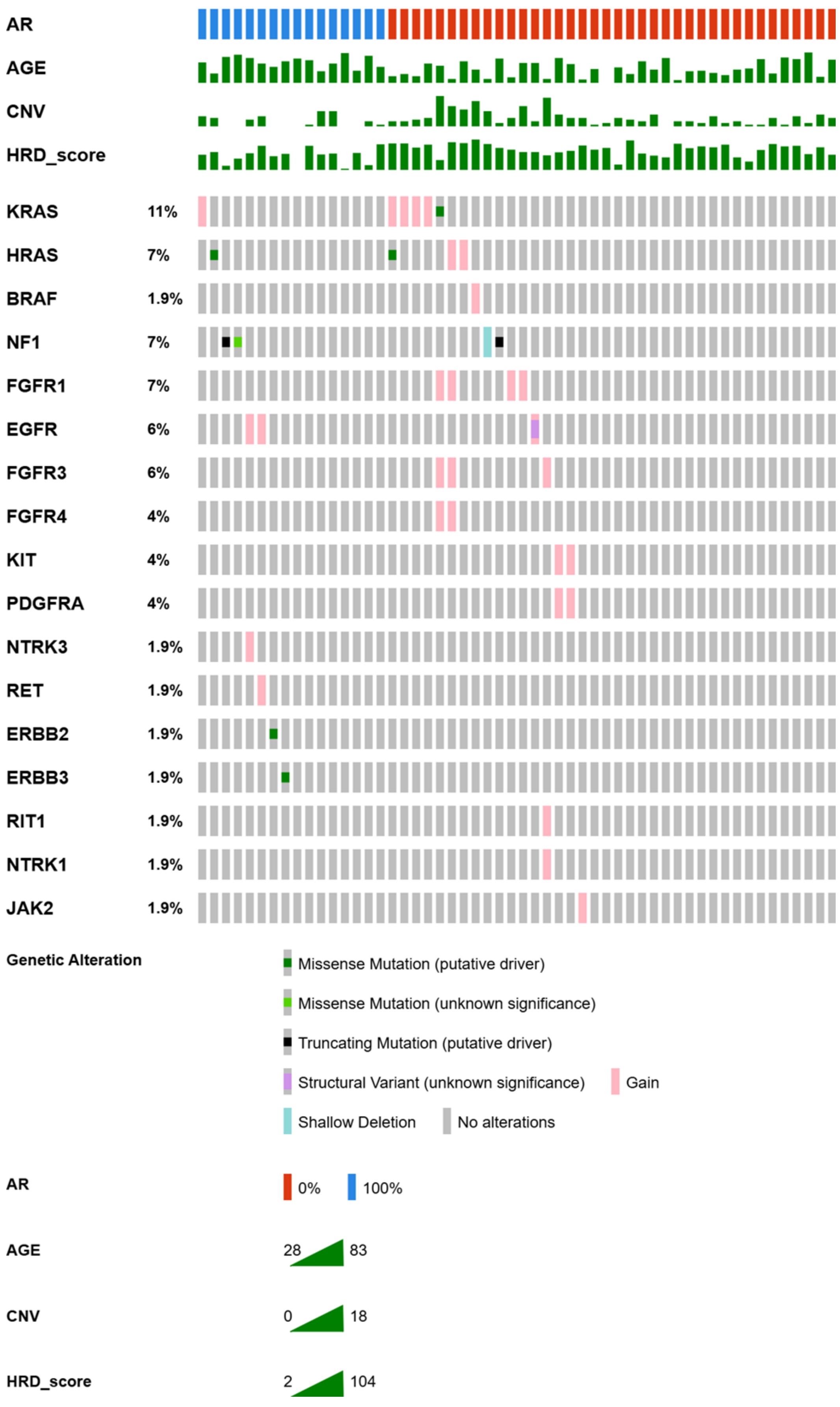
AR
AGE
CNV
HRD_score
KRAS 11%
HRAS 7%
BRAF 1.9%
NF1 7%
FGFR1 7%
EGFR 6%
FGFR3 6%
FGFR4 4%
KIT 4%
PDGFRA 4%
NTRK3 1.9%
RET 1.9%
ERBB2 1.9%
ERBB3 1.9%
RIT1 1.9%
NTRK1 1.9%
JAK2 1.9%
Genetic Alteration
Missense Mutation (putative driver)
Missense Mutation (unknown significance)
Truncating Mutation (putative driver)
Structural Variant (unknown significance)
Gain
Shallow Deletion
No alterations
AR
0%
100%
AGE
28
83
CNV
0
18
HRD_score
2
104

(a)

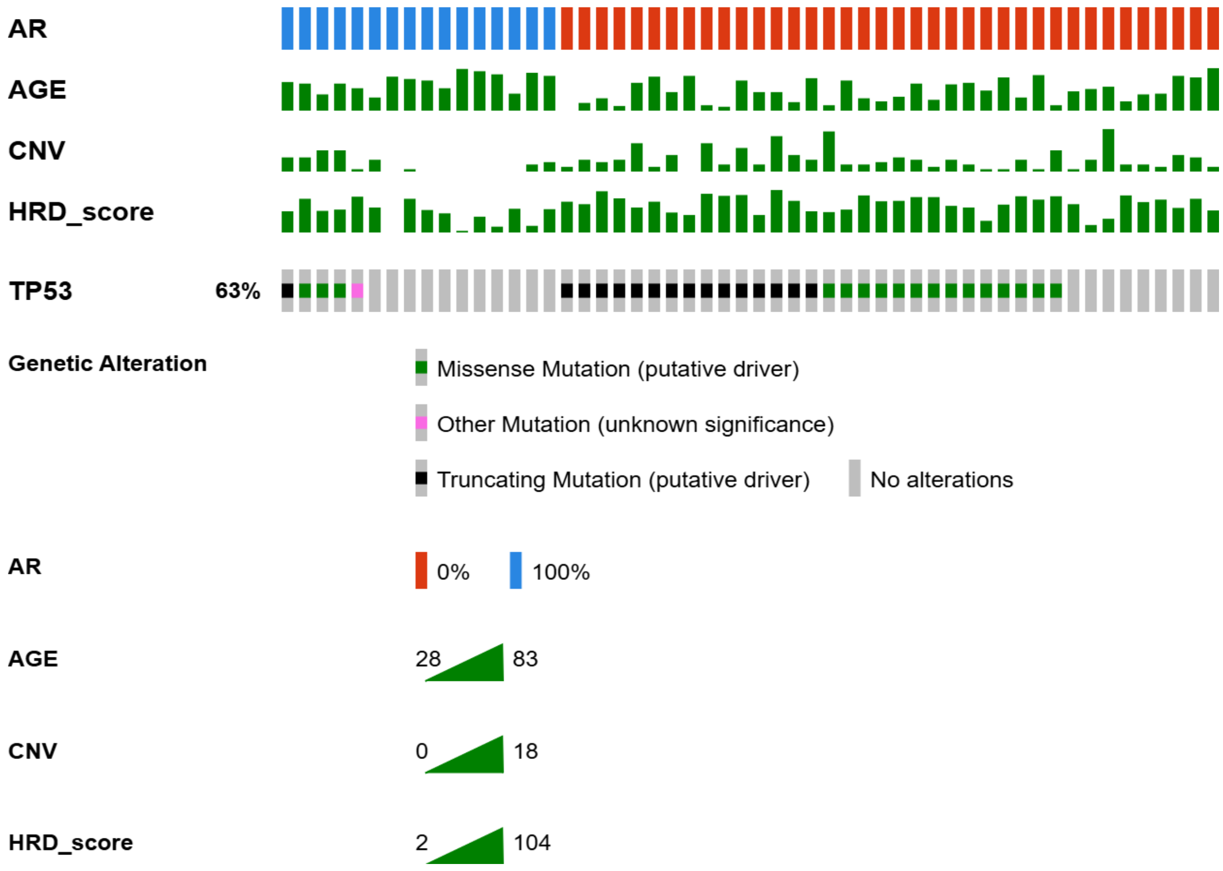
AR
AGE
CNV
HRD_score
TP53
63%
Genetic Alteration
Missense Mutation (putative driver)
Other Mutation (unknown significance)
Truncating Mutation (putative driver)
No alterations
AR
0%
100%
AGE
28
83
CNV
0
18
HRD_score
2
104

(**b**)

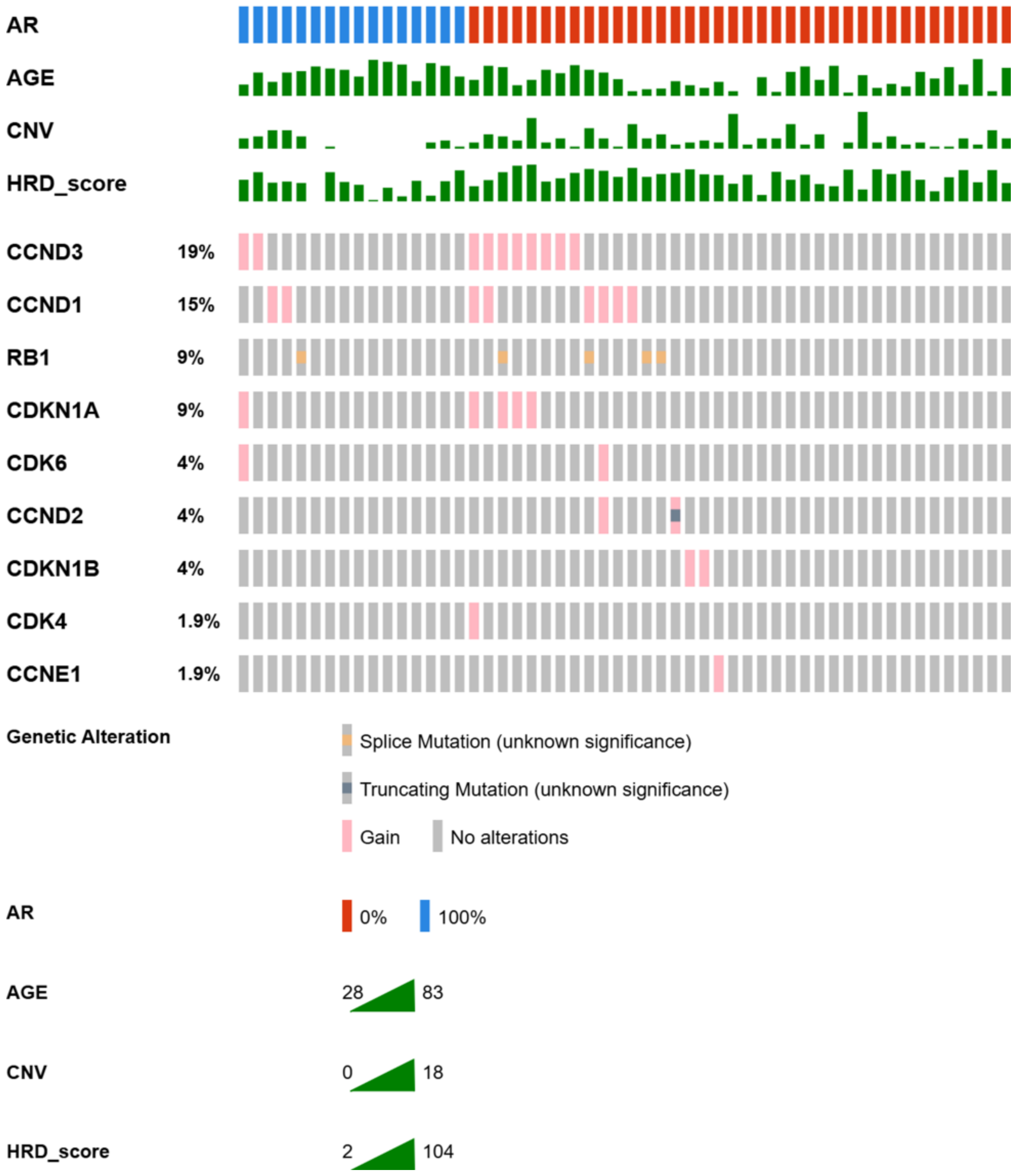
AR
AGE
CNV
HRD_score
CCND3 19%
CCND1 15%
RB1 9%
CDKN1A 9%
CDK6 4%
CCND2 4%
CDKN1B 4%
CDK4 1.9%
CCNE1 1.9%
Genetic Alteration
Splice Mutation (unknown significance)
Truncating Mutation (unknown significance)
Gain
No alterations
AR
0%
100%
AGE
28
83
CNV
0
18
HRD_score
2
104

(c)

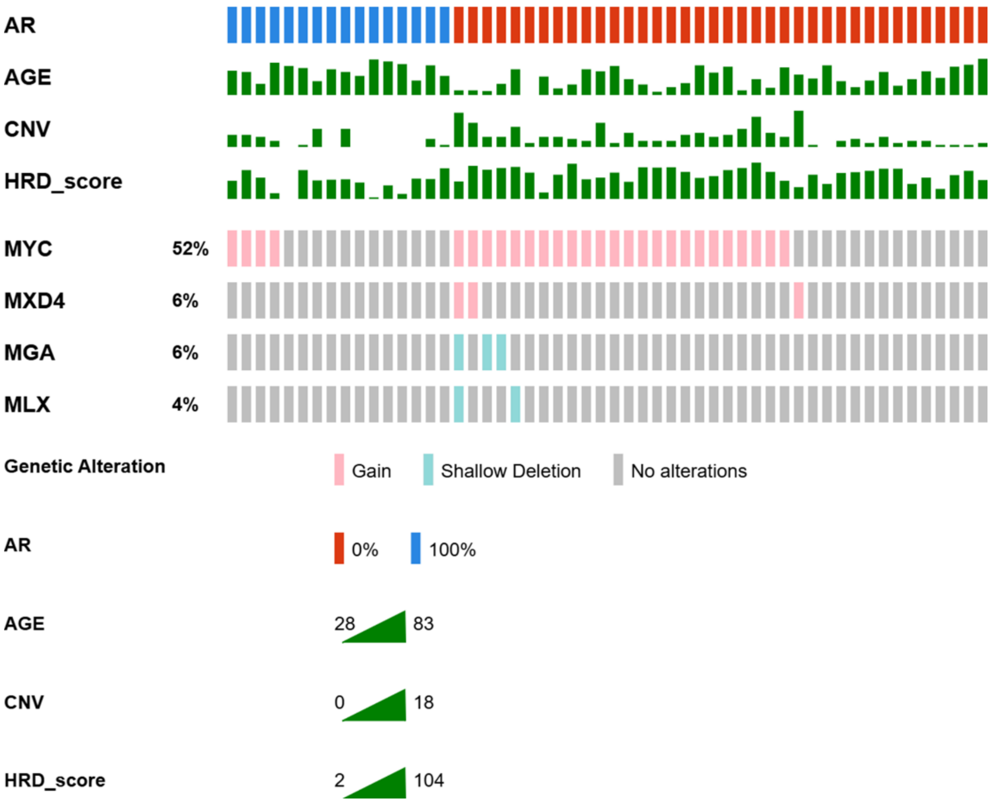
AR
AGE
CNV
HRD_score
MYC 52%
MXD4 6%
MGA 6%
MLX 4%
Genetic Alteration
Gain
Shallow Deletion
No alterations
AR
0%
100%
AGE
28
83
CNV
0
18
HRD_score
2
104

(**d**)

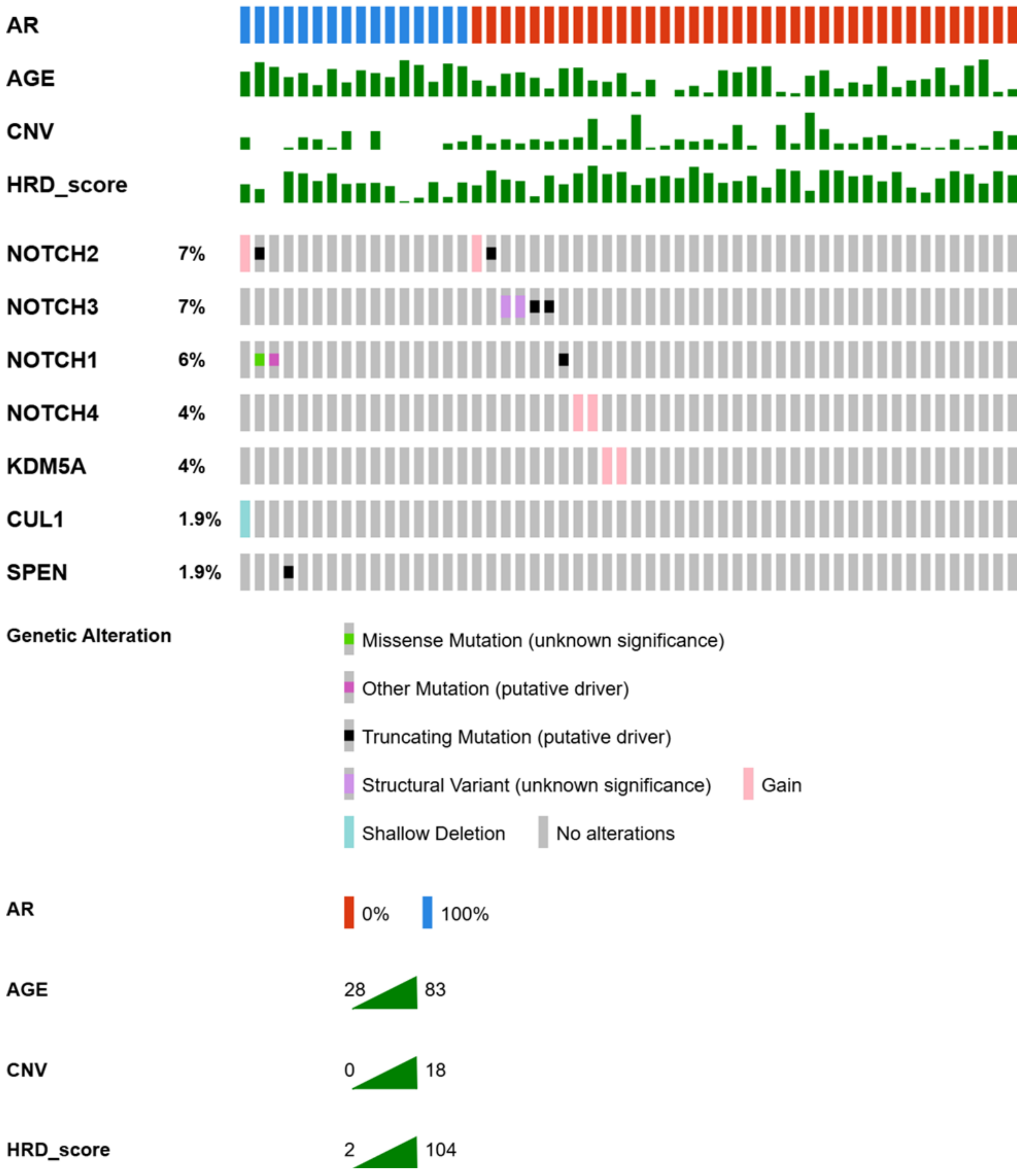
AR
AGE
CNV
HRD_score
NOTCH2 7%
NOTCH3 7%
NOTCH1 6%
NOTCH4 4%
KDM5A 4%
CUL1 1.9%
SPEN 1.9%
Genetic Alteration
Missense Mutation (unknown significance)
Other Mutation (putative driver)
Truncating Mutation (putative driver)
Structural Variant (unknown significance)
Gain
Shallow Deletion
No alterations
AR
0%
100%
AGE
28
83
CNV
0
18
HRD_score
2
104

(e)

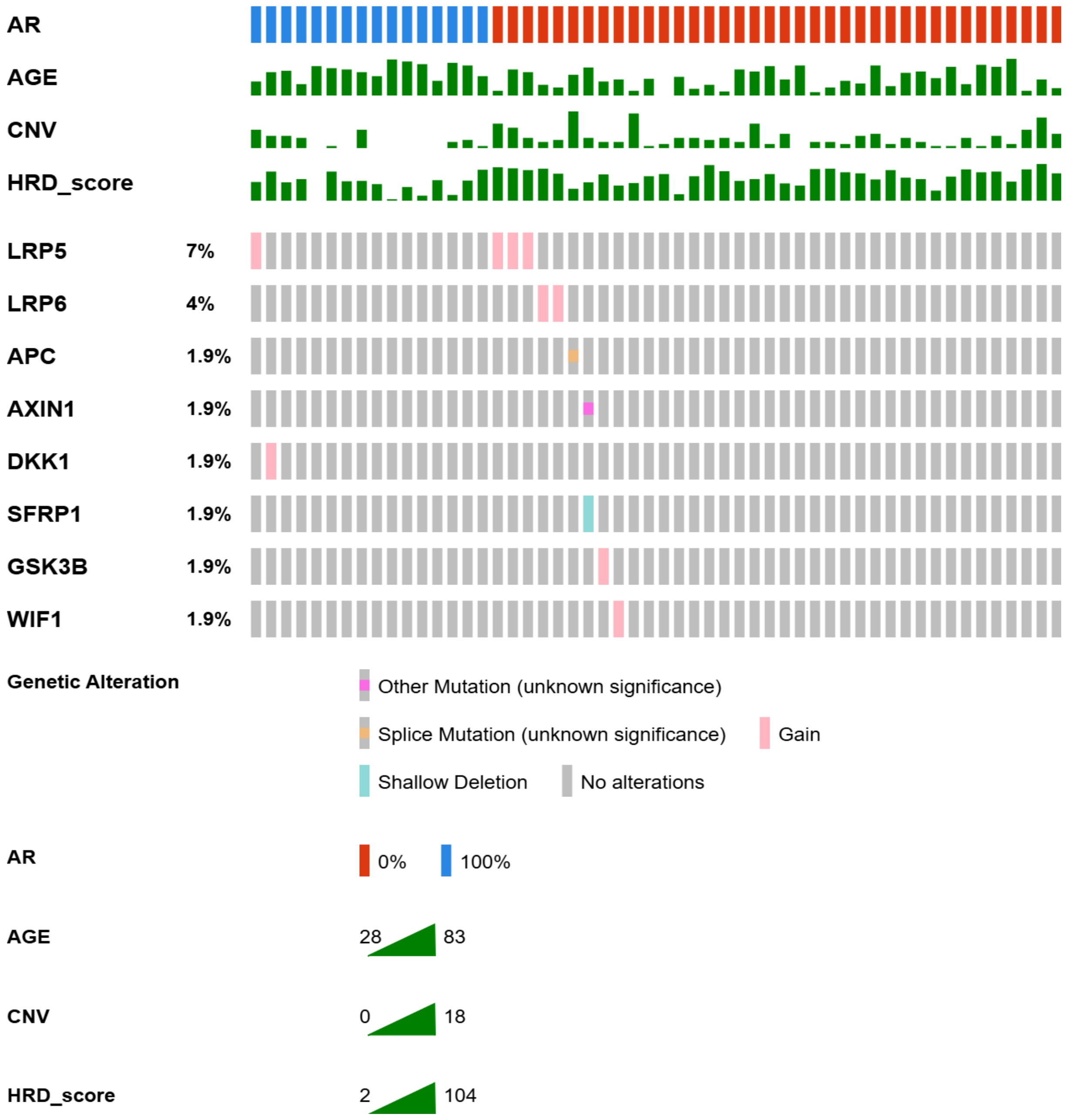

(f)

Figure S1: Alterations in pathogenic/likely pathogenic (P/LP) variants proportions according to AR status in TNBCs main cancer related pathways in (a) RTK/RAS pathway, (b) TP53, (c) Cell cycle pathway, (d) MYC pathway, (e) NOTCH pathway and (f) WNT signaling pathway.
